# Transvaginal small bowel evisceration after cervical loop electrosurgical excision procedure: a case report

**DOI:** 10.1186/s12905-023-02650-3

**Published:** 2023-09-19

**Authors:** Kui Yao, Chuan Xie

**Affiliations:** 1 Department of Gynecology and Obstetrics, West China Second University Hospital, Sichuan University, Chengdu, Sichuan Province P.R. China; 2 Key Laboratory of Birth Defects and Related Diseases of Women and Children, Ministry of Education, Chengdu, Sichuan Province P.R. China; 3 Department of Gynecology and Obstetrics, Key Laboratory of Birth Defects and Related Diseases of Women and Children, Ministry of Education, West China Second University Hospital, Sichuan University, No. 20 Section Three, South Renmin Road, Chengdu, Sichuan Province 610041 P.R. China

**Keywords:** Evisceration, Transvaginal, Small bowel, Loop electrosurgical excision procedure, Case report

## Abstract

Transvaginal small bowel evisceration is a life-threatening condition, which is usually seen in postmenopausal women with a history of gynecological surgery. Cervical loop electrosurgical excision procedure (LEEP) is widely used in the treatment of cervical intraepithelial neoplasia (CIN), and its surgical risk and postoperative complications are relatively low because of the simplicity of the operation. However, improper operation may result in perforation of the uterus, which can cause prolapse of the small bowel into the vagina. We here reported an extremely rare case of a young woman with transvaginal small bowel evisceration after cervical LEEP, achieving a good prognosis after the prolapsed bowel was reduced. The patient underwent cervical LEEP as a treatment for CIN III, but the LEEP resulted in a laceration of about 4.0 cm × 3.5 cm on the peritoneum of the uterovesical peritoneal reflection and a laceration of about 2.0 cm × 1.5 cm on the anterior wall of the cervical canal. Through the two lacerations, the pelvic cavity is connected to the vagina and the small intestine prolapsed into the vagina. Due to aggressive surgical intervention, the patient achieved a favorable prognosis after successfully reducing the prolapsed bowel.

## Introduction

Transvaginal small bowel evisceration is a rare but potentially life-threatening condition that requires urgent surgical intervention to prevent intestinal necrosis. It often occurs when vaginal cuff dehiscence after hysterectomy, with high-risk factors including old age, serious pelvic-organ prolapse (POP), and a history of transvaginal surgery [1]. Timely identification and surgical intervention are the two most important factors in the management of transvaginal small bowel evisceration. Cervical loop electrosurgical excision procedure (LEEP) is a widely used, safe, and effective surgical method for the treatment of cervical intraepithelial neoplasia (CIN), and to the best of our knowledge, there have been no reports of transvaginal small bowel evisceration after cervical LEEP in English or Chinese literature [2]. Hence, we first reported a case of acute small bowel evisceration into the vagina and incarceration after cervical LEEP.

## Case report

**Fig. 1:**
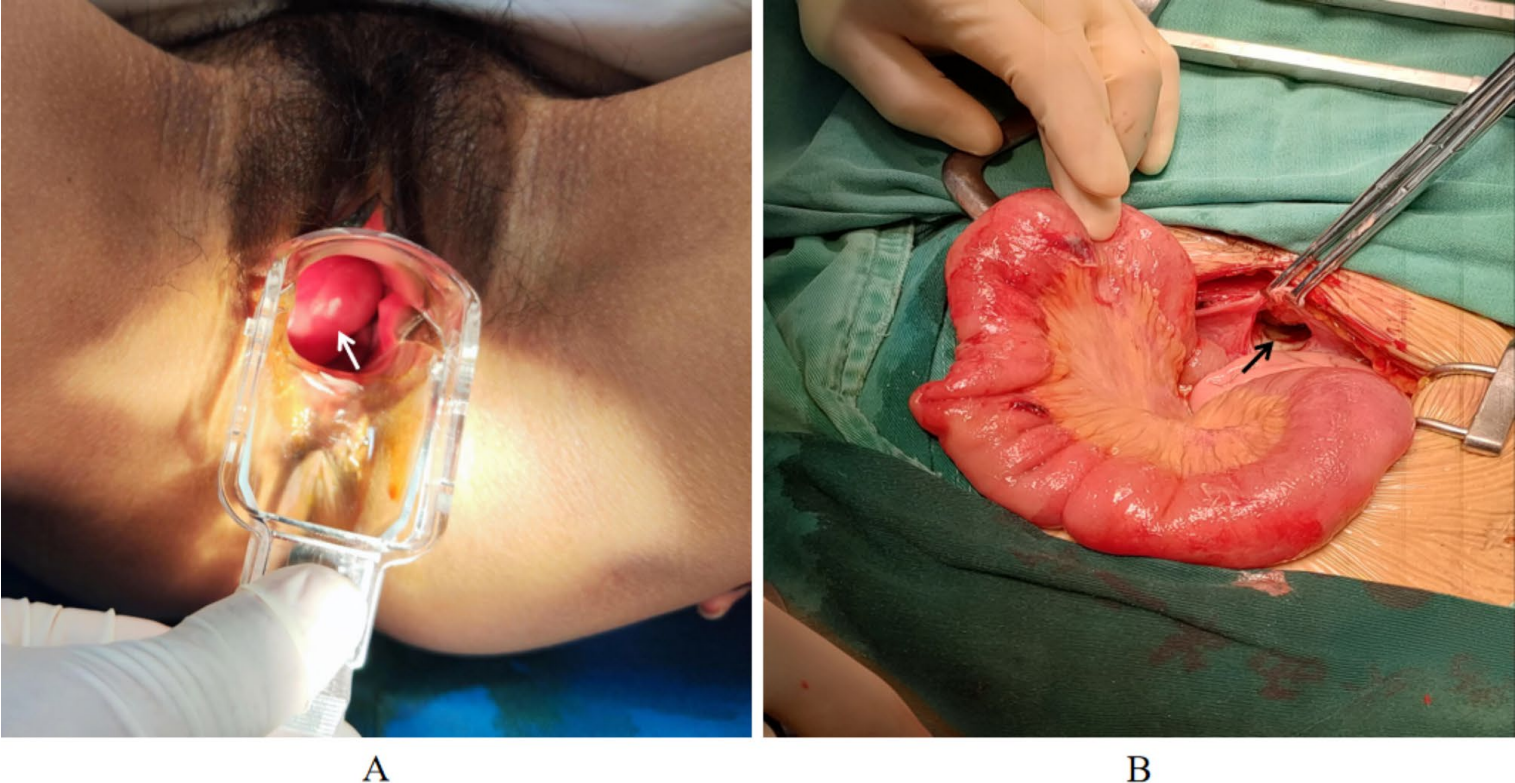
**A**, a prolapse of the small bowel was found through vaginal speculum inspection. The white arrow points to the small bowel. **B**, Intraoperative findings showed a laceration of about 4.0 cm×3.5 cm on the peritoneum of the uterovesical peritoneal reflection and a laceration of about 2.0 cm × 1.5 cm on the anterior wall of the cervical canal. The black arrow points to the laceration on the peritoneum of the uterovesical peritoneal reflection

A 31-year-old female patient received cervical LEEP on December 7, 2022, due to CIN grade II. Due to active bleeding after surgery, a piece of gauze was inserted into her vagina to stop the bleeding. The patient presented to the emergency department in the evening of the same day complaining of mild lower abdominal pain, accompanied by slight nausea and vomiting. The emergency gynecologist considered that these discomfort were normal post-operative reaction, so no special treatment and vaginal examination were carried out. Due to the aggravation of abdominal pain, the patient presented to the emergency department again on the second day after cervical LEEP. The patient had two previous normal vaginal deliveries without any surgical history. It was her first cervical surgery, and pathological diagnosis of the cervix after LEEP revealed CIN Grade III. Physical examination revealed her abdominal swelling and diffuse tenderness, but her vital signs were stable, without any signs of infection such as fever or a high number of white blood cells. Gynecological ultrasound showed no abnormality, but emergency abdominal CT examination indicated intestinal obstruction. After removing the gauze in her vagina, a prolapse of the small bowel was found through vaginal speculum inspection (Fig. 1A). Based on the above findings, the presence of small intestine incarceration in the vagina was highly suspected, so an emergency exploratory laparotomy was performed. Intraoperative findings showed a laceration of about 4.0 cm×3.5 cm on the peritoneum of the uterovesical peritoneal reflection and a laceration of about 2.0 cm × 1.5 cm on the anterior wall of the cervical canal (Fig. 1B). The small intestine prolapsed into the vagina through the above two lacerations. Fortunately, the serosal layer of the small bowel was intact and ruddy, without marked ischemia and necrosis, and the bladder was not damaged. After the prolapsed bowel was reduced, the subsequent exploration revealed that the local tissues around the two openings were edema and brittle, accompanied by pus mosses, and the opening on the anterior wall of the cervical canal was connected to the vagina. The absorbable suture was used in the surgery to repair the wound on the anterior wall of the cervical canal and the peritoneum of the uterovesical peritoneal reflection.

The patient’s postoperative course was uneventful, and she was discharged 7 days after surgery without any immediate postoperative complications. She has been following up in the gynaecology outpatient department since the surgery.

## Discussion

Transvaginal small bowel evisceration is a rare surgical condition first described by Hypernaux et al. in 1864. Nearly 100 cases have been reported so far, all of which occurred after total hysterectomy, and more than 70% of them are postmenopausal women. Transvaginal small bowel evisceration is mainly related to a history of vaginal surgery, enterocele, and other factors, which may be related to decreased vaginal wall vascularity and atrophy of the vaginal wall in post-menopausal women [3, 4]. In premenopausal women, it is associated with sexual intercourse and vaginal trauma.

Cervical LEEP, a widely used outpatient electrosurgical treatment, emerged in the 1990s, using a 30 ~ 40 W high-frequency electric knife for the treatment of cervical lesions. Complications of LEEP include bleeding, infection, cervical adhesion, cervical insufficiency during pregnancy, and rare cervical or uterine perforation [5]. The perforation from the anterior cervical wall to the uterine bladder peritoneal reflection is extremely rare, which can lead to bowel evisceration.

The cervical LEEP instrument used in this case was an electrified wire loop. We speculated that the excision was deep in the process of resection of the anterior lip of the cervix, and the patient’s uterus was backward flexion, thus leading to the perforation from the anterior cervical wall to the peritoneum of the uterovesical peritoneal reflection. Apart from the deep resection site, there was also heat damage and subsequent local inflammation, which contributed to the peritoneal perforation. Due to the burning wound and other factors affecting surgical field exposure during cervical LEEP, it is difficult to be detected in time, which laid a hidden danger for the subsequent small bowel prolapse.

Patients with transvaginal evisceration of the intra-abdominal organs often presented with pelvic or vaginal pain, vaginal mass, or visible lumps between the legs, and more often occur in the vaginal cuff dehiscence. The distal ileum is the most common protruding viscera, although other organs such as epiploon, and fallopian tubes have also been described. Small bowel prolapse may be complicated by the insufficient blood supply to the bowel, and then intestinal ischemia and necrosis [6]. In this case, the anterior cervical wall has a smaller wound area and more dense surrounding tissue, and the probability of intestinal necrosis and intestinal obstruction symptoms will also increase.

Transvaginal small bowel evisceration is a surgical emergency, and there is a certain risk of death, about 6 to 8%. Once the transvaginal small bowel evisceration is found, the prolapsed bowel should be reduced urgently. If the small bowel is not damaged and the vaginal elasticity is good, it can be reduced trans-vaginally, but laparotomy was more recommended [4]. In this case, the scope of the opening in the anterior cervical wall was small, and the elasticity of the tissues around the electrical cauterization was poor, accompanied by pus mosses and the adhesion of the intestinal tube, so laparotomy was adopted. The incidence of small bowel damage is up to 15 ~ 20%, which is related to the occurrence time and degree of prolapse. Bowel resection needs to be performed on about 20% of patients [7]. The intestinal blood supply and activity should be carefully investigated, and the gynecologists and surgeons should work together.

## Conclusion

The safety and effectiveness of cervical LEEP have been widely recognized, but doctors should still pay attention to the resection angle and avoid too deep resection, especially in the uterus in a special position or patients with a history of cervical surgery. When there are unusual symptoms such as severe abdominal pain after LEEP, a routine vaginal examination should be performed to find rare cases such as small bowel prolapse in time. Early detection and surgical management of small bowel prolapse are necessary to reduce the risk of resection of intestinal necrosis, which may progress to sepsis, systemic inflammatory response syndrome, and ultimately death.

## Data Availability

The datasets used and/or analysed during the current study available from the corresponding author on reasonable request.

## Abbreviations

CIN: Cervical intraepithelial neoplasia
LEEP: Loop electrosurgical excision procedure
